# Imaging and Quantification of mRNA Molecules at Single-Cell Resolution in the Human Fungal Pathogen Candida albicans

**DOI:** 10.1128/mSphere.00411-21

**Published:** 2021-07-07

**Authors:** Sergio D. Moreno-Velásquez, J. Christian Pérez

**Affiliations:** a Interdisciplinary Center for Clinical Research, University Hospital Würzburg, Würzburg, Germany; b Institute for Molecular Infection Biology, University Würzburg, Würzburg, Germany; c Department of Microbiology and Molecular Genetics, McGovern Medical School, The University of Texas Health Science Center at Houston, Houston, Texas, USA; University of Georgia

**Keywords:** hybridization chain reaction, FISH, *Candida albicans*, mRNA, single-cell analysis

## Abstract

The study of gene expression in fungi has typically relied on measuring transcripts in populations of cells. A major disadvantage of this approach is that the transcripts’ spatial distribution and stochastic variation among individual cells within a clonal population is lost. Traditional fluorescence *in situ* hybridization techniques have been of limited use in fungi due to poor specificity and high background signal. Here, we report that *in situ* hybridization chain reaction (HCR), a method that employs split-initiator probes to trigger signal amplification upon mRNA-probe hybridization, is ideally suited for the imaging and quantification of low-abundance transcripts at single-cell resolution in the fungus Candida albicans. We show that HCR allows the absolute quantification of transcripts within a cell by microscopy as well as their relative quantification by flow cytometry. mRNA imaging also revealed the subcellular localization of specific transcripts. Furthermore, we establish that HCR is amenable to multiplexing by visualizing different transcripts in the same cell. Finally, we combine HCR with immunostaining to image specific mRNAs and proteins simultaneously within a single C. albicans cell. The fungus is a major pathogen in humans where it can colonize and invade mucosal surfaces and most internal organs. The technical development that we introduce, therefore, paves the way to study the patterns of expression of pathogenesis-associated C. albicans genes in infected organs at single-cell resolution.

**IMPORTANCE** Tools to visualize and quantify transcripts at single-cell resolution have enabled the dissection of spatiotemporal patterns of gene expression in animal cells and tissues. Yet the accurate quantification of transcripts at single-cell resolution remains challenging for the much smaller microbial cells. Widespread phenomena such as stochastic variation in transcript levels among cells—even within a clonal population—seem to play important roles in the biology of many microorganisms. Investigating this process requires microbial cell-optimized procedures to image and measure mRNAs at single-molecule resolution. In this report, we adapt and expand *in situ* hybridization chain reaction (HCR) combined with split-initiator probes to visualize transcripts in the human-pathogenic fungus Candida albicans at high resolution.

## INTRODUCTION

Gene expression analysis is most often carried out with RNA samples derived from large pools of cells. In such measurements, the abundance of any given transcript reflects the “average” expression across thousands or millions of cells. Transcript level variation among individual cells—a rampant phenomenon observed in populations of clonal cells from all domains of life (1–3)—is simply not recorded with such approaches. Likewise, the spatial distribution of transcripts (within a cell as well as across cells in three-dimensional structures such as biofilms or tissues) is lost in these analyses. Recently developed single-cell RNA sequencing methodologies have overcome many of these shortcomings in animal cells (4, 5). While important strides have been made with single-cell RNA sequencing in the model yeast Saccharomyces cerevisiae and a few bacteria as well (6–8), the accurate quantification of transcripts at single-cell resolution remains challenging for unicellular organisms. Furthermore, single-cell RNA sequencing does not provide information on the subcellular distribution of transcripts.

*In situ* hybridization has been widely used to visualize transcripts within cells, tissues, organs, and organisms (9, 10). However, conventional *in situ* hybridization strategies remain cumbersome, multiplexed experiments are impractical, the signal readout depends on either colorimetric or fluorescent enzymatic methods which are limited by low signal-to-noise ratio, and products are precipitates that usually diffuse away from the probes (11, 12). Novel fluorescence *in situ* hybridization (FISH) methods with increased sensitivity have been developed. For example, single-molecule fluorescence *in situ* hybridization (smFISH) relies on the use of multiple fluorescent oligonucleotides that hybridize to a target RNA (12, 13). Although smFISH has proven to be sensitive enough to simultaneously detect several mRNA molecules, the synthesis of the probes is rather burdensome, and the method is limited because it requires highly specialized instruments (e.g., microscope with inclined laser illumination) (12, 14). In fungi in particular, fluorescence *in situ* hybridization techniques have been of limited use due to poor specificity and high background signal.

*In situ* hybridization based on the mechanism of the hybridization chain reaction (HCR), which is used in animal cells, offers a sensitive and specific method to visualize transcripts in single cells (15, 16). HCR is an isothermal enzyme-free nucleic acid-based amplification method that enables the detection of specific DNA sequences (17). The amplification step involves the continuous binding of two complementary hairpins (amplifiers) that upon exposure to a specific DNA sequence (initiator) yields 10- to 100-fold signal enhancement (15, 18). Moreover, a recent improvement to the method (HCR v3.0) introduced the use of split-initiator probes which results in automatic background suppression, thus significantly increasing the signal-to-background ratio (19). HCR has enabled state-of-the-art mRNA imaging at high resolution in animal cells, tissues, and whole embryos (16).

The fungus Candida albicans is a pathobiont in the human intestine as well as oral and vaginal mucosae. Cell-to-cell mRNA variability as well as the specific subcellular localization of transcripts seems to drive important aspects of the biology of this organism. For example, C. albicans displays cell-to-cell transcript variability in *EFG1*, a regulator that is critical for intestinal colonization (20). On the other hand, transcripts with key roles in C. albicans yeast-to-filament transition and virulence are transported to specific subcellular compartments (21, 22). Tools for the analysis of *Candida* transcripts at single-cell resolution, therefore, can stimulate and expand research in these areas. Here, we describe a simple and robust method to image and quantify mRNAs at single-cell resolution in C. albicans based on HCR combined with split-initiator probes. We demonstrate that this method is well suited for the visualization and quantification of low-abundance transcripts. Furthermore, we show that HCR is amenable to multiplexing and to combining with immunostaining to image multiple transcripts and proteins simultaneously. The protocol that we outline can be readily applied to other fungi.

## RESULTS

### Rationale.

The scarcity of well-described protocols to image and quantify mRNAs at single-cell resolution in *Candida*—and more broadly in human fungal pathogens—motivated us to search for current FISH-related procedures being employed in other experimental organisms. Two C. albicans features make this organism particularly challenging to image with traditional FISH probes at the resolution needed to distinguish single transcripts: its hard-to-permeabilize cell wall and high autofluorescence levels. HCR, which was developed for animal cells, offered an alternative explicitly designed to deal with samples with high fluorescence background. Earlier HCR procedures (v2.0) relied on the use of multiple long probes (50 nucleotides [nt]), each carrying two full HCR initiators (16). Nonspecific binding of the probe within the sample can trigger HCR, resulting in amplified background (23, 24). In order to reduce the background caused by either the nonspecific binding of probes or other autofluorescent molecules (e.g., vitamins), we combined HCR with the use of split-initiator probes (25 nt each) which automatically suppresses background upon nonspecific hybridization (HCR v3.0) (19).

### Imaging and quantification of mRNAs encoding C. albicans regulatory proteins.

**FIG 1:**
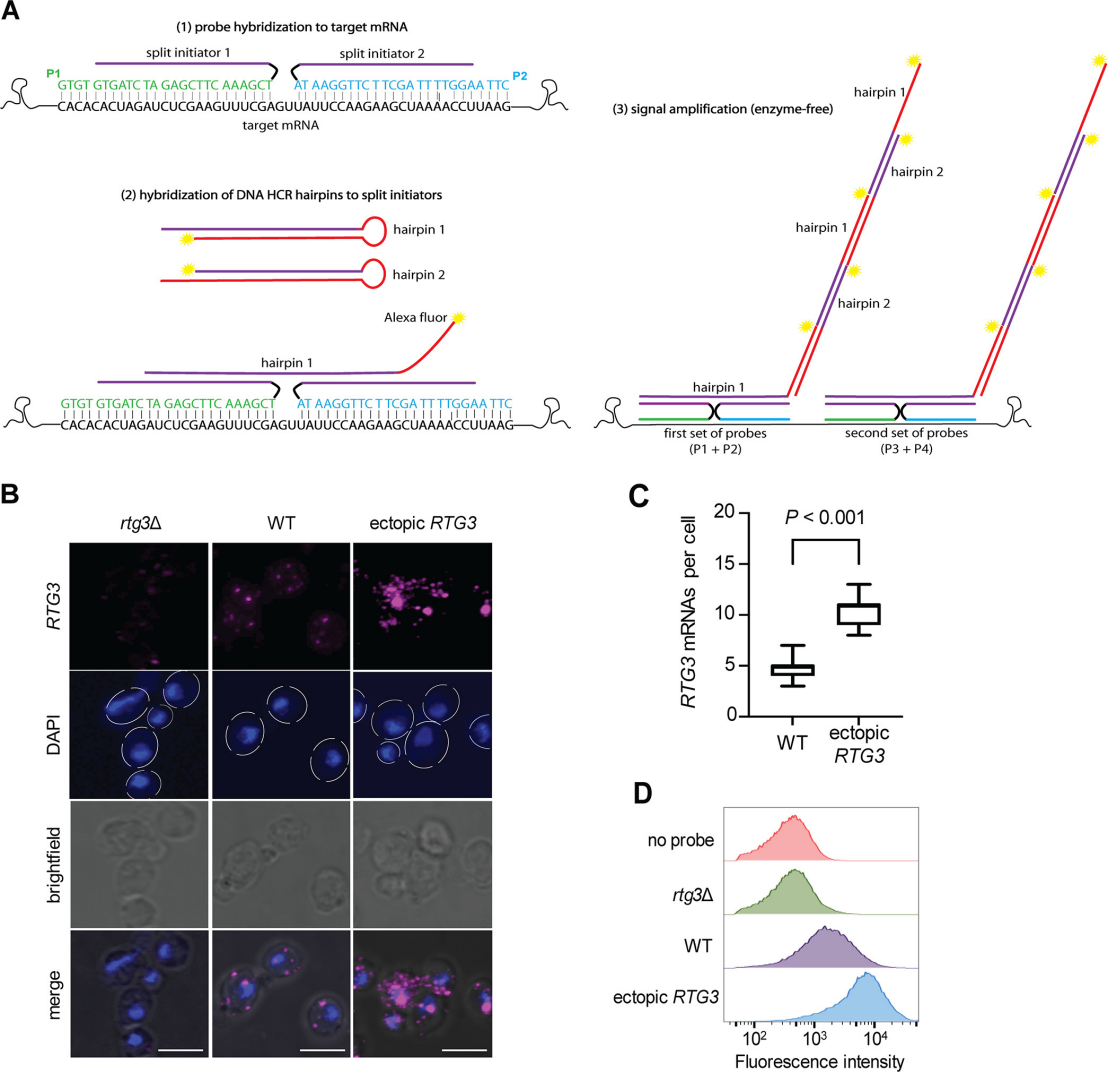
Hybridization chain reaction (HCR) enables the visualization and quantification of transcripts at single-cell resolution in C. albicans. (A) Schematic of HCR. Split-initiator probes are designed to target specific mRNAs. Two probes hybridize to a target mRNA at adjacent sites (separated by 2 nt). Each probe contributes half of the initiator sequence. The initial hairpin amplifier (labeled with a fluorophore) exposes a new initiator resulting in signal amplification. (B) High-resolution confocal laser scanning microscopy imaging of the *RTG3* transcript—which encodes a transcription factor—in the indicated C. albicans strains (WT, wild type). Hairpins were conjugated with Alexa Fluor 647 (magenta). Nuclei were stained with DAPI (blue). Images are maximal Z-stack projections where the blue, magenta, and bright-field channels are shown separate and merged. Bars, 5 μm. (C) Quantification of *RTG3* transcripts. The mean (horizontal line in the middle of the box) is plotted with interquartile range (box) and the 10th to 90th percentile (error bars) (*n *= 90 cells per strain). Statistical analysis was performed by Student’s *t* test. (D) Relative quantification of the *RTG3* transcript by flow cytometry. A total of 50,000 cells were evaluated per sample. The “no probe” control measures the background fluorescence of the fungal cells.

A schematic depicting the design of probes and the HCR principle is shown in Fig. 1A. We designed up to 10 pairs of reverse complement probes (hereafter termed split probes) for each transcript of interest. Each pair of split probes carries half of the HCR initiator sequence (18 nt) that is complementary to the HCR hairpins. Only the split probe pairs that specifically hybridize adjacent to each other to the transcript of interest allow the hybridization of fluorescently labeled complementary HCR hairpins. The specific interaction between split-initiator probes and hairpins triggers an amplification chain reaction while automatically suppressing the background produced by nonspecific probe binding.

We first sought to visualize and quantify C. albicans transcripts encoding regulatory proteins, which typically are expressed at low abundance (25, 26). We chose *RTG3* and *TYE7* which encode two transcription regulators that govern sphingolipid homeostasis and carbohydrate metabolism, respectively, and are key components of the regulatory network driving C. albicans commensalism and pathogenicity (27–29). To stringently evaluate whether the fluorescent signal corresponded to the transcripts of interest, we probed each mRNA in three corresponding strains, the wild type, deletion mutant, and a strain in which the gene of interest was under the control of the strong promoter *TDH3*.

Images of C. albicans cells in which the *RTG3* transcript was stained are shown in Fig. 1B (corresponding images for *TYE7* are included in Fig. S1 in the supplemental material). The wild-type strain displayed discrete punctae (4.92 ± 1.49 per cell) (Fig. 1C) randomly distributed throughout the cytoplasm. Each “dot” most likely represents a single transcript molecule. The fluorescent signal originated specifically in the *RTG3* transcript because the *rtg3* deletion strain displayed no comparable staining. In some preparations, traces of fluorescent signal were found at the bottom of the slide, likely due to the nonspecific amplification of deposited material that had not been washed away. As expected, cells in which *RTG3* transcription was driven by the strong promoter *THD3* displayed increased signal: 11.2 ± 1.86 discrete punctae per cell (Fig. 1C) and overall higher fluorescence intensity. Furthermore, the majority of cells ectopically expressing *RTG3* displayed a strong “dot” of fluorescent signal in close proximity to the nucleus, likely corresponding to the spatial accumulation of >1 *RTG3* mRNA molecules. Similar patterns of staining (in the wild type, *tye7* deletion mutant, and a strain ectopically expressing *TYE7*) were observed for the *TYE7* transcript as well (Fig. S1).

10.1128/mSphere.00411-21.5 FIG S1 HCR visualization of the C. albicans transcript *TYE7*, which encodes a transcription factor. Shown are confocal microscopy images of C. albicans yeast cells processed following the HCR procedure. Hairpins were conjugated with Alexa Fluor 488 (green). (A) WT; (B) *tye7* deletion mutant; (C) strain in which *TYE7* transcription is driven by the strong promoter *TDH3*. Zoomed-in images corresponding to the dashed squares are shown in the panels to the right. Bars, 5 μm. Download FIG S1, TIF file, 2.1 MB. Copyright © 2021 Moreno-Velásquez and Pérez. 2021 Moreno-Velásquez and Pérez. https://creativecommons.org/licenses/by/4.0/ This content is distributed under the terms of the Creative Commons Attribution 4.0 International license.

We next wanted to establish whether the fluorescent signal generated by the HCR procedure in C. albicans is sufficient to carry out relative quantifications by flow cytometry. As shown in Fig. 1D, the fluorescence intensity measured by flow cytometry closely resembled our microscopy observations. For example, the population of cells ectopically expressing *RTG3* produced, on average, higher signal than the population of wild-type cells. Moreover, the fluorescence intensity measured in the *rtg3* deletion strain was similar to that of the unstained wild-type strain (the latter cells were exposed to hairpins but not probes). This shows the high specificity of HCR and split-initiator probes even when transcripts are present in relatively small amounts.

**FIG 2:**
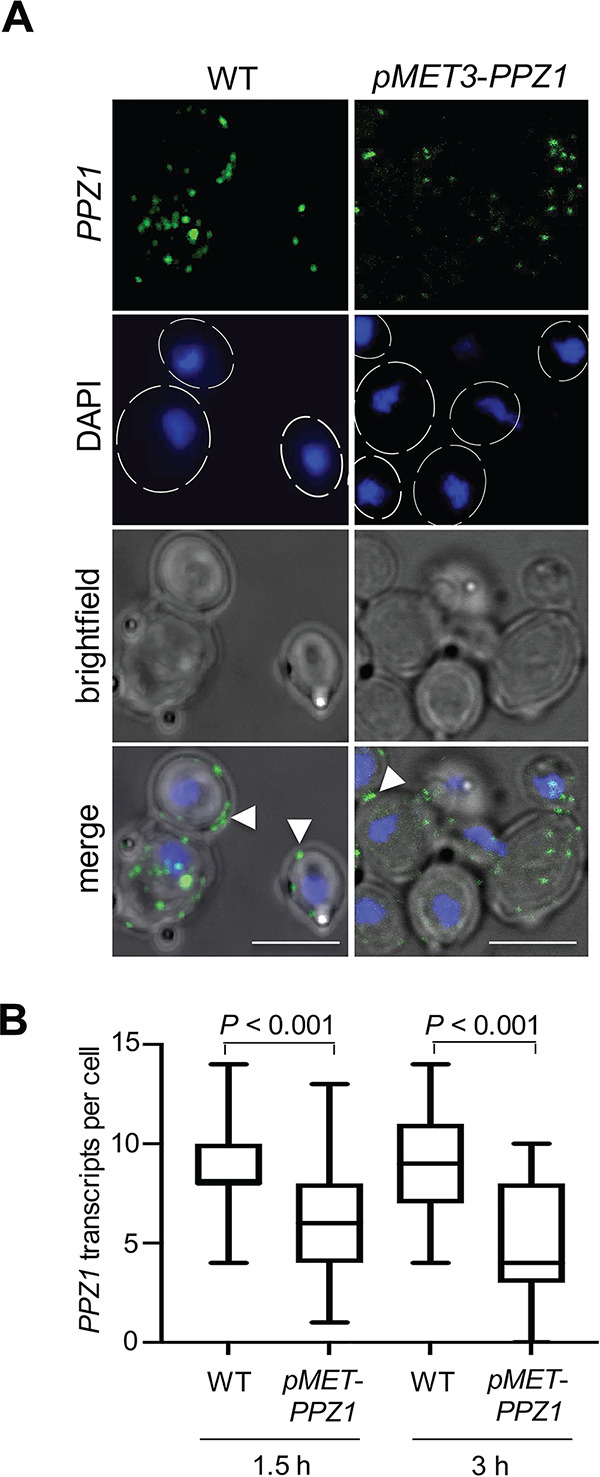
Visualization and quantification of the *PPZ1* transcript that encodes a protein phosphatase. (A) High-resolution confocal imaging of the *PPZ1* transcript in wild-type (WT) cells and in a strain in which *PPZ1* expression has been placed under the control of the repressible promoter *MET3*. Strains were grown at 37°C for 1.5 and 3 h in YNB medium containing cysteine and methionine (2.5 mM). Hairpins were conjugated with Alexa Fluor 488 (green). Nuclei were stained with DAPI (blue). Images are maximal Z-stack projections where the blue, green, and bright-field channels are shown separate as well as merged. The arrowheads in the bottom panels indicate transcripts adjacent to cell membrane. Bars, 5 μm. (B) Transcript quantification by confocal laser scanning microscopy. The mean (horizontal line in the middle of the box) is plotted with the interquartile range (box) and the 10th to 90th percentile (error bars) (*n *= 30 cells per strain per time point). Statistical analysis was performed by the Student’s *t* test.

Another transcript selected for visualization and quantification in C. albicans was *PPZ1*, which encodes a fungus-specific serine/threonine phosphatase involved in cation homeostasis, cell wall integrity, and virulence (30). As a complementary strategy to the overexpression of *RTG3* and *TYE7* described above, here we included a C. albicans strain in which *PPZ1* expression had been placed under the control of the repressible promoter *MET3* (31). In this strain, *PPZ1* transcription initiation can be effectively reduced by the addition of the amino acids methionine and cysteine (32). Our microscopy analysis and quantification in wild-type cells revealed 8.8 ± 2 *PPZ1* transcripts per cell (assuming each discrete “dot” of fluorescent signal corresponds to a single transcript molecule) (Fig. 2A and B). A significant fraction of *PPZ1* transcripts did not seem randomly distributed in the cytoplasm but rather localized adjacent to the cell membrane (Fig. 2A and Table 1). As expected, upon addition of methionine and cysteine to repress *PPZ1* expression, the number of *PPZ1* transcripts was significantly reduced (to 6.03 ± 2 transcripts per cell after 1.5 h and further to 4.8 ± 2.4 transcripts per cell after 3 h) (Fig. 2B). We noticed that, despite the reduction in transcript number, the subcellular localization of the *PPZ1* mRNAs remained unchanged (i.e., they still localized adjacent to the cell membrane) (Table 1).

**TABLE 1:** Distribution of *PPZ1* transcripts within C. albicans yeast cells

Strain	Treatment	No. of transcripts^*a*^	
		In cytoplasm (not close to cell membrane)	Adjacent to cell membrane
WT		5 ± 1.3*	3.8 ± 0.7
*pMET-PPZ1*	1.5-h inhibition	3.93 ± 1.48*	2.1 ± 0.52
	3-h inhibition	3.12 ± 1.42*	1.68 ± 0.98

a Values are shown as means ± SD (*n *= 30 cells per strain per treatment). *, *P* < 0.05 (compared to transcripts adjacent to cell membrane).

We also designed probes to visualize the transcript *PGK1* which encodes the metabolic enzyme phosphoglycerate kinase (33). As for all the other transcripts described above, we observed clear intracellular punctae upon staining with the specified probes in wild-type cells (Fig. S2). The use of either only probes or only hairpins resulted in no detectable signal (Fig. S2), further validating the specificity of the split-initiator probes and overall HCR method. Altogether, our results demonstrate that combining HCR with split-initiator probes is suitable to image and quantify transcripts at single-cell resolution in C. albicans.

10.1128/mSphere.00411-21.6 FIG S2 Combining HCR with split initiators in C. albicans results in specific transcript amplification. Shown are confocal microscopy images of C. albicans cells hybridized with hairpins conjugated to Alexa Fluor 488 (green). HCR components added as follows: (A) only hairpins; (B) only split-initiator probes; (C) neither probes nor hairpins; (D) hairpins plus split initiator probes designed to detect the phosphoglycerate kinase *PGK1* transcript. Notice that background signal was not subtracted. Bars, 5 μm. Download FIG S2, TIF file, 1.5 MB. Copyright © 2021 Moreno-Velásquez and Pérez. 2021 Moreno-Velásquez and Pérez. https://creativecommons.org/licenses/by/4.0/ This content is distributed under the terms of the Creative Commons Attribution 4.0 International license.

### mRNA subcellular localization.

**FIG 3:**
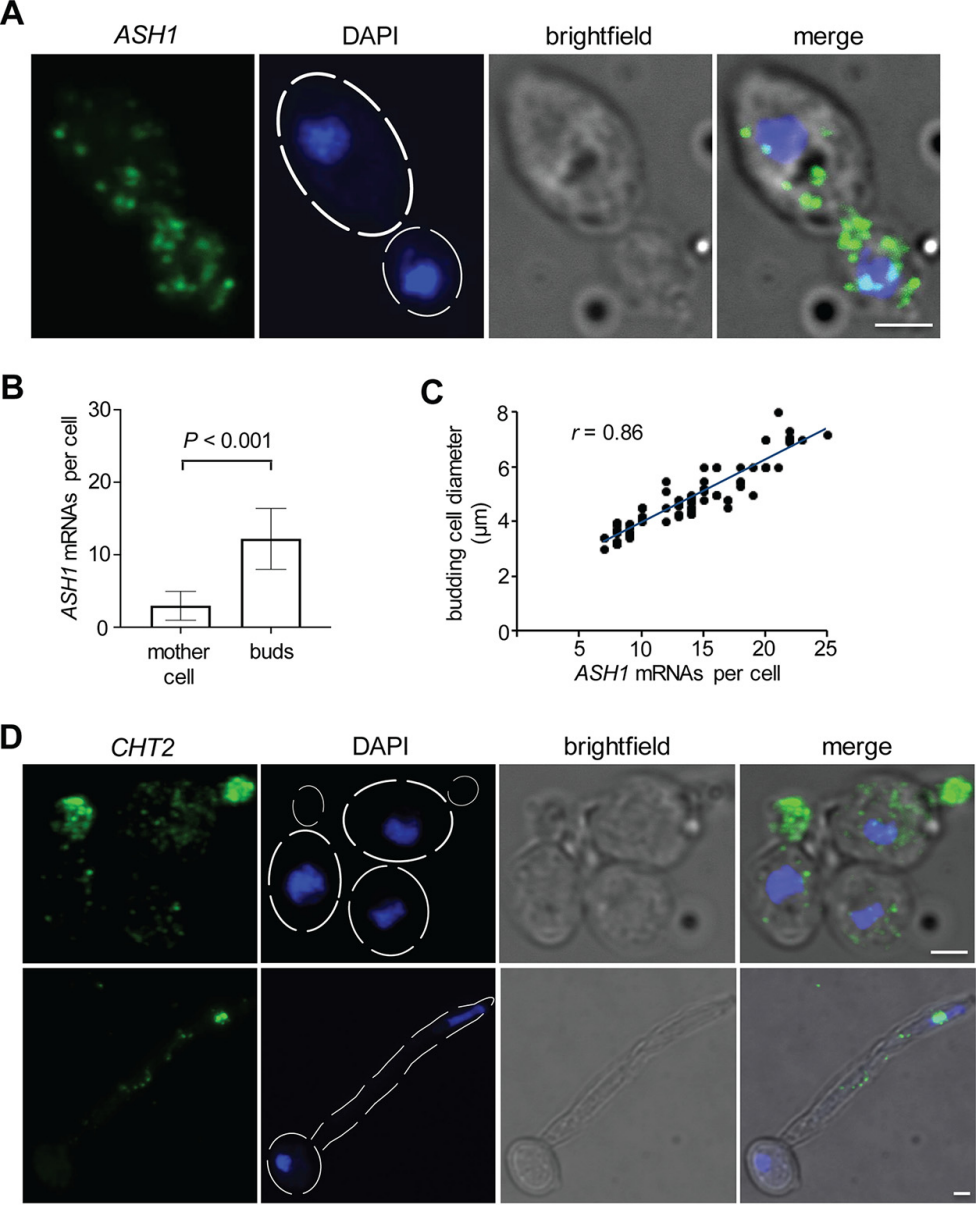
Establishing the subcellular distribution of transcripts in C. albicans. (A) High-resolution confocal laser scanning microscopy images to visualize the *ASH1* transcript. (B) Quantification of *ASH1* mRNAs in mother and budding daughter cells indicates a clear preference for the latter. The means ± SD (error bars) are plotted (*n *= 30 cells.). Statistical analysis was performed by Student’s *t* test. (C) Number of *ASH1* mRNAs as a function of budding cell size. Each dot represents a single cell (*n *= 35). The trend line is indicated in blue. Pearson’s correlation coefficient (*r*) is included. (D) High-resolution confocal laser scanning microscopy images to visualize the *CHT2* transcript that encodes a chitinase. The images in the top row show yeast cells, whereas the images in the bottom row depict the distribution in hyphae. The *CHT2* transcript localizes preferentially in budding daughter cells or toward the distal end of the filament. Hairpins were conjugated with Alexa Fluor 488 (green). Nuclei were stained with DAPI (blue). Images are maximal Z-stack projections. The blue, green, and bright-field channels are shown separate and merged. Bars, 2 μm.

The localization of specific transcripts to different parts of the cell is an important mechanism to achieve polarity and asymmetric growth (14). FISH has been instrumental to study the subcellular localization of mRNAs in many organisms. We reasoned that HCR may be a particularly powerful tool for this purpose in C. albicans. As proof of principle, we chose to visualize two mRNAs that have been shown to localize to particular compartments in C. albicans (22): the mRNA encoding the transcriptional regulator Ash1p and the mRNA encoding the GPI-linked chitinase Cht2p. The implementation of HCR with split-initiator probes for these two transcripts resulted in high-quality, nitid images with clearly discernible punctae in both the round “yeast” form of the fungus and in hyphae (Fig. 3). In agreement with a previous report (22), the *ASH1* and *CHT2* transcripts primarily localized to the daughter cells during budding (Fig. 3A, B, and D). We measured daughter cell size and counted the number of transcripts present per cell (Fig. 3C). This analysis revealed a positive correlation between the diameter of the daughter cells and the amount of *ASH1* transcript (Fig. 3C), which may reflect the cell cycle dependence of *ASH1* bud localization (22).

We also imaged the *CHT2* mRNA in hyphae (Fig. 3D, bottom panel). The transcripts were found along the filament but showed a higher concentration in the apical zone near the tip. The basal cell (where the filament emerges by polarized growth) seemed, for the most part, depleted of *CHT2* transcripts.

### Visualization of multiple transcripts in the same cell (multiplexing).

A drawback in conventional *in situ* hybridization is the difficulty of multiplexing (i.e., detecting more than one mRNA target in each sample). Typically, a serial amplification for each particular target is needed, which results in significant increases in processing time when analyzing >1 transcript (34, 35). Another common problem is the loss of resolution resulting from the diffusion of reporter molecules prior to deposition, which leads to blurry spots (i.e., unclear transcript boundaries) (36). HCR can circumvent these longstanding challenges by the use of orthogonal hairpins that can independently recognize specific initiator sequences upon hybridization with the mRNA target (15).

**FIG 4:**
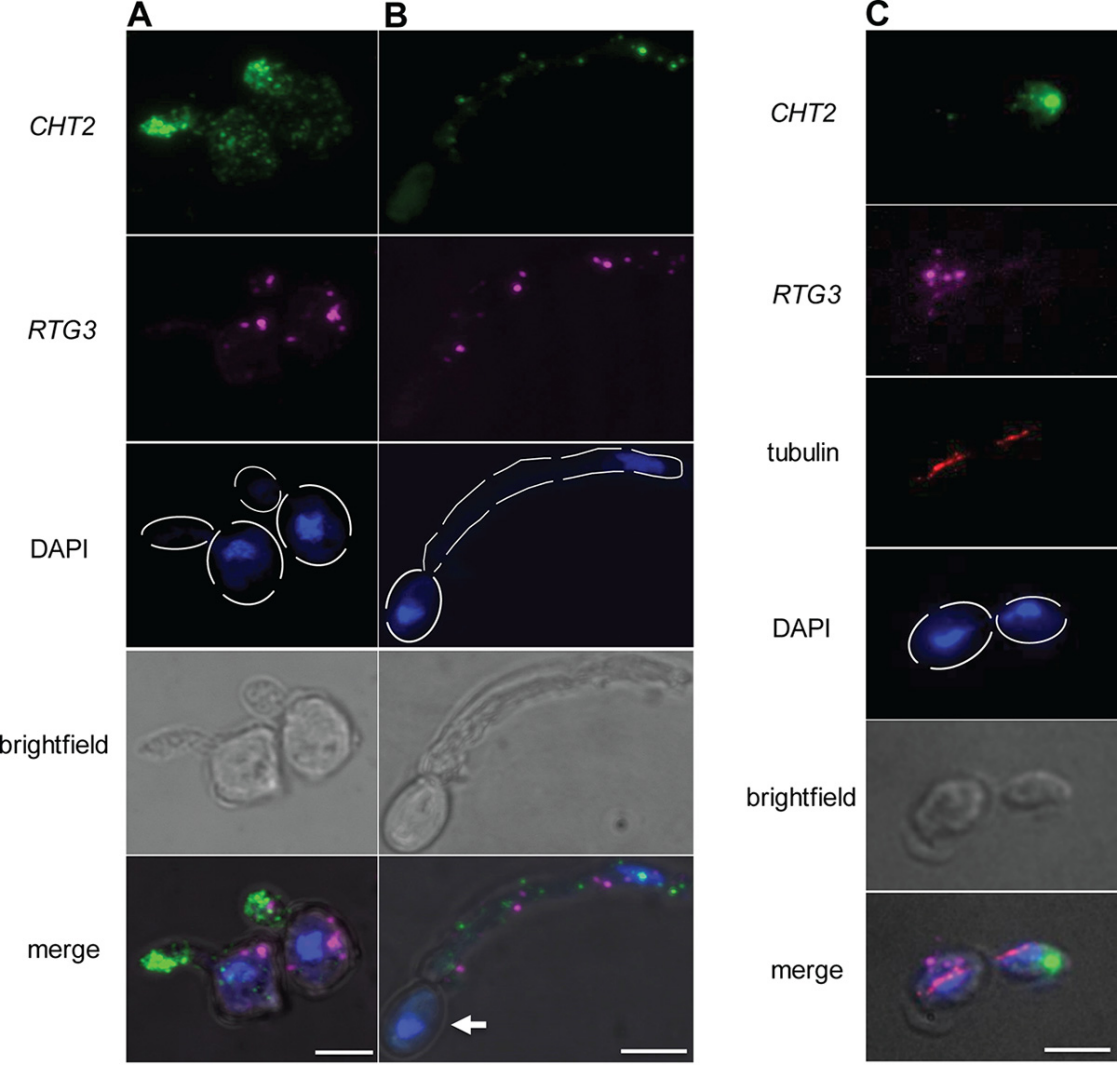
Detection of multiple transcripts simultaneously in multiplex HCR and imaging mRNAs together with proteins by coimmunostaining. High-resolution confocal imaging using hairpins conjugated to either Alexa Fluor 488 or Alexa Fluor 647. Nuclei were stained with DAPI (blue). *CHT2* (green), and *RTG3* (magenta) transcripts in yeast cells (A) or hyphae (B). Notice the lack of transcripts in the basal compartment (white arrow) that gives rise to the hypha. (C) Combined HCR and immunostaining to simultaneously detect the transcripts *CHT2* and *RTG3* in addition to the protein ɑ-tubulin (red). Images are maximal Z-stack projections. Images corresponding to the blue, green, and bright-field channels are shown separate and merged. Bars, 5 μm.

To simultaneously visualize >1 mRNA target in the same C. albicans cell, we coincubated our samples with amplification hairpins B2 and B3 (see Materials and Methods). High-resolution confocal microscopy allowed the clear visualization of both *RTG3* and *CHT2* transcripts in the same cell, both in yeast (Fig. 4A) and hyphae (Fig. 4B). We also costained in the same cell the *RTG3* and *TYE7* mRNAs (Fig. S3A). The coincubation with multiple hairpins did not affect the localization of the signal corresponding to either transcript (compare Fig. 4A to Fig. 1B and 3A where only one hairpin was used). Despite the use of 40 split-initiator probes (20 probes for each mRNA target), we still detected discrete punctae with differential localization and undetected cellular background. It was also possible to examine three mRNA targets (using a total of 60 split-initiator probes) in a single cell, although with reduced resolution between transcripts (Fig. S3B).

10.1128/mSphere.00411-21.7 FIG S3 Simultaneous detection of multiple transcripts by HCR in C. albicans. Shown are high-resolution confocal images using hairpins conjugated with Alexa Fluor 488, Alexa Fluor 647, and Alexa Fluor 546. Nuclei were stained with DAPI (blue). (A) *RTG3* (magenta) and *TYE7* (green) transcripts. (B) *RTG3* (magenta), *CHT2* (green) and *ASH1* (red) transcripts. Bars, 5 μm. Download FIG S3, TIF file, 1.9 MB. Copyright © 2021 Moreno-Velásquez and Pérez. 2021 Moreno-Velásquez and Pérez. https://creativecommons.org/licenses/by/4.0/ This content is distributed under the terms of the Creative Commons Attribution 4.0 International license.

We were able to directly quantify the number of transcripts per hyphal cell (Fig. 4B), a process limited in flow cytometry measurements due in part to the inherent tendency of hyphae to aggregate (37). Spot counting by microscopy revealed that hyphae contain 16 ± 3 and 7 ± 2 transcripts for *CHT2* and *RTG3*, respectively. We notice that in hyphae, both *CHT2* and *RTG3* transcripts were found in the filament, but not in the basal cell (Fig. 4B). These observations indicate that multiple C. albicans transcripts preferentially localize to the active growth area of the filament.

### Combining HCR with immunostaining.

Finally, we sought to combine multiplex HCR with conventional immunodetection to simultaneously visualize specific transcripts and proteins in C. albicans. For this, we first performed the HCR amplification steps for the transcripts *CHT2* and *RTG3* followed by immunostaining of the cytoskeleton protein α-tubulin. We optimized the procedures to achieve reduced incubation times that can be carried out at room temperature (see Materials and Methods). As shown in Fig. 4C, with the optimized method we were able to obtain images in which both transcripts as well as α-tubulin could be readily visualized (Fig. 4C). The fluorescent signal corresponding to both transcripts and the α-tubulin protein remained high, whereas the cytoplasmic background was kept low (enough signal remained in the samples for imaging even after 2 weeks of storage in darkness at 4°C). As expected (14), the *CHT2* mRNA localized almost exclusively in the budding daughter cell, whereas α-tubulin extended from the mother to the daughter cell (38). Thus, although the overall resolution of the images was somewhat reduced, the subcellular localization of the mRNAs and the tubulin was not altered by the procedure. This method, therefore, constitutes a robust and reliable approach to simultaneously evaluate the localization of transcripts and proteins of interest in C. albicans.

## DISCUSSION

In this report, we have adapted and expanded the *in situ* hybridization chain reaction (HCR) technology to a fungus. Specifically, we demonstrate that the combination of split-initiator probes and HCR enables the visualization—in unprecedented detail—and quantification of mRNAs at various levels of expression in C. albicans. The remarkably high signal-to-noise ratio achieved in our procedure made transcript quantification possible using not only microscopy but also flow cytometry. The orthogonal hairpin amplifiers that we describe are compatible with the sample processing steps that had to be optimized or added to the workflow to deal with particular features of fungal cells such as removal of the cell wall. Furthermore, we show that up to three different mRNAs could be imaged simultaneously within a single yeast cell. Altogether, the described methodology is likely to facilitate and stimulate mRNA imaging studies in pathogenic fungi.

Traditional methods of *in situ* hybridization to visualize transcripts have relied on the use of long self-made probes in combination with catalytic reporter deposition. Such an approach has several drawbacks. (i) Synthetic, long probes penetrate into cells with reduced efficiency. (ii) The probes are highly prone to degradation. (iii) The use of antibodies generates high levels of background. (iv) Typically, a single transcript can be detected in each sample. (v) The results are qualitative rather than quantitative. (vi) The spatial resolution is compromised by diffusion of the reporter molecules (22, 36, 39–42). While improved versions of the FISH method, e.g., single-molecule FISH, are amenable to multiplexing, they still rely on the use of multiple oligonucleotides (10 to 50 nt long) per mRNA target. Critical to the success of smFISH is the design of optimal probes, a nontrivial process that has to take into account parameters such as base composition and secondary structure formation (12, 43). In contrast to smFISH, extensive probe optimization is not required in HCR. The HCR strategy employed here requires ∼15 to 20 oligonucleotides. The combinatorial use of split-initiator probes automatically suppresses background (19) and significantly increases the signal-to-noise ratio. In fact, the signal enhancement that we achieved in C. albicans cells makes the method suitable for imaging using objectives with low numerical apertures (see Fig. S3 in the supplemental material) and quantification employing flow cytometry, which enables high-throughput single-cell studies. Furthermore, the visualization of mRNAs simultaneously with the cytoskeleton protein α-tubulin (Fig. 4C) demonstrate that HCR can be used in combination with immunostaining to monitor the spatial distribution of proteins and specific transcripts at high resolution in fungal cells. HCR and immunostaining have been combined before in murine retina sections, although the imaging in those studies was limited to low resolution (44).

We successfully stained and visualized a diverse set of transcripts in C. albicans, including the mRNAs encoding the transcriptional regulators Rtg3p, Tye7p (Fig. S1), and Ash1p as well as the transcripts encoding the protein phosphatase Ppz1, the phosphoglycerate kinase Pgk1p (Fig. S2), and the chitinase Cht2p. These transcripts were imaged at single-cell resolution in both yeast and hyphal forms of the fungus. The procedure allowed us to determine whether the evaluated mRNAs localized preferentially to certain compartments (e.g., body of the hyphae, mother or daughter cells). We noticed, for example, that the *RTG3* and *CHT2* transcripts localized to the body of the hyphae (Fig. 4B), suggesting that during hyphal development, the distribution of several transcripts may be restricted to active areas of cell growth to allow efficient local translation at the site of protein function (45). A similar protein localization tendency toward the active growing filament is observed in filamentous fungi (46–48). It is thought that, in these organisms, the proteins are transported toward the mature tip of the filament by the polarization machinery.

There are still several challenges and limitations to the application of *in situ* hybridization chain reaction technology in fungal cells. (i) Although nonspecific binding of individual probes did not result in any appreciable amplified background, the nonspecific binding to other reagents or to the surface of the coverslip may generate some amplified background. (ii) The relative quantification by flow cytometry in a large number of cells becomes challenging when the transcript levels are low (approximately one to four copies per cell) or are distributed in the body of the hyphae (filaments tend to clump, making flow cytometry unworkable). (iii) Multiplexed experiments result in reduced resolution of single transcripts, particularly when using epifluorescence microscopy. (iv) The experimental workflow remains complex for high-throughput studies.

The approach described in this report opens a new state-of-the-art cell-imaging avenue to study gene expression in fungi. We anticipate that the described methodology will be particularly useful to researchers interested in exploring the spatiotemporal patterns of expression of specific genes in medically important fungal pathogens. For example, *Candida*-colonized mammalian tissues are typically highly heterogeneous, suggesting that individual *Candida* cells may execute different expression programs depending on the particular microenvironment surrounding them. The high levels of specific signal amplification and reduction in background achieved with the HCR strategy make it an ideal candidate to visualize the distribution of pathogenesis-associated transcripts in fungal cells located in host tissues. Such imaging experiments are likely to reveal key information on hallmarks of the infection site and its topography that correlate with the expression of fungal genes of interest. These studies remain largely missing in the field of fungal, and more broadly, microbial pathogenesis.

## MATERIALS AND METHODS

### Strains and growth conditions.

All C. albicans strains were derivatives of the clinical isolate SC5314 and are listed in Table S1 in the supplemental material. C. albicans was routinely grown at 30°C in yeast-peptone-dextrose (YPD) liquid medium (10 g yeast extract, 20 g peptone, and 20 g dextrose per liter) in a shaking incubator. C. albicans transformants were selected on YPD agar supplemented with nourseothricin (200 μg/ml) (Jena Bioscience). All plasmids were maintained in Escherichia coli DH5α which was grown at 37°C in Luria-Bertani (Miller) (LB) medium supplemented with the appropriate antibiotic (100 μg/ml).

10.1128/mSphere.00411-21.1 TABLE S1 Strains used in this study. Download Table S1, PDF file, 0.03 MB. Copyright © 2021 Moreno-Velásquez and Pérez. 2021 Moreno-Velásquez and Pérez. https://creativecommons.org/licenses/by/4.0/ This content is distributed under the terms of the Creative Commons Attribution 4.0 International license.

### Strain construction.

Plasmids and oligonucleotides are listed in Tables S2 and S3, respectively. Gene deletions in C. albicans were carried out with the *HIS*/*FLP* system for marker/CRISPR-Cas9 recycling (49). Briefly, cloning-free guide RNA (gRNA) cassette stitching to target the *RTG3* gene was assembled by fusion PCR. The Cas9 expression plasmid was digested with FastDigest MssI (Thermo Scientific). Synthetic donor DNAs were designed as complementary oligonucleotides (100 bp in length, with 50 bp flanking homology upstream and downstream of the open reading frame [ORF]) and annealed in 1× FastDigest buffer. After transformation and selection, removal of CRISPR components was performed by growing colony-purified isolates overnight in YP-maltose. The gRNA was designed on Benchling (San Francisco, CA).

10.1128/mSphere.00411-21.2 TABLE S2 Plasmids used in this study. Download Table S2, PDF file, 0.02 MB. Copyright © 2021 Moreno-Velásquez and Pérez. 2021 Moreno-Velásquez and Pérez. https://creativecommons.org/licenses/by/4.0/ This content is distributed under the terms of the Creative Commons Attribution 4.0 International license.

10.1128/mSphere.00411-21.3 TABLE S3 Oligonucleotides used in this study. Download Table S3, XLSX file, 0.01 MB. Copyright © 2021 Moreno-Velásquez and Pérez. 2021 Moreno-Velásquez and Pérez. https://creativecommons.org/licenses/by/4.0/ This content is distributed under the terms of the Creative Commons Attribution 4.0 International license.

To generate the heterozygous *PPZ1*/*ppz1*Δ strain, one copy of the gene was removed using the *SAT1* flipper as described previously (50). Briefly, ∼500 nt upstream and downstream of the *PPZ1* ORF were PCR amplified with primer pairs JCP_2716/2912 and JCP_2718/2719, respectively. The amplified products were introduced in the KpnI/ApaI and SacII/SacI sites of the *SAT1* flipper cassette (pSFS5) to generate plasmid JCP_1112. The KpnI/SacI fragment of plasmid JCP_1112 was used for transformation in C. albicans. Colony-purified and fully vetted (i.e., confirmed by both colony PCR and Sanger sequencing) transformants were grown overnight in YCB-BSA-YE medium (23.4 g yeast carbon base [YCB], 4 g bovine serum albumin [BSA], 2 g yeast extract [YE] per liter [pH 4.0]) to remove the *SAT1* flipper cassette. Subsequently, the endogenous promoter of the remaining *PPZ1* copy was replaced with the repressible *MET3* promoter (31). For this, PCR was used to amplify the *SAT1* flipper cassette (primers JCP_2898/2899) and the promoter *MET3* (primers JCP_2900/2901) from plasmids pADH34 and pADH33, respectively. The resulting PCR products were used as the templates for fusion PCR (51) with nested primers JCP_2902/2903. The final DNA fragment was used for C. albicans transformation by electroporation. Colony-purified and fully vetted transformants were grown overnight in YP-maltose to remove the *SAT1* flipper cassette. Correct modification of the *PPZ1* promoter was confirmed by colony PCR with primers JCP_2904/2905 and Sanger sequencing.

Unless indicated otherwise, genomic DNA from C. albicans SC5314 was used as the template for all PCRs. Phusion high-fidelity DNA polymerase (New England Biolabs) was employed to amplify all DNA fragments for cloning or for direct transformation into C. albicans. *Taq* DNA polymerase with ThermoPol buffer (New England Biolabs) was used for colony PCR and strain vetting.

### Design of split-initiator probes.

Target DNA sequences were obtained from the *Candida* Genome Database (http://www.candidagenome.org/). The split-initiator probes were purchased as standard desalted DNA oligonucleotides from Sigma-Aldrich (Germany). All DNA oligonucleotides employed in HCR are listed in Table S4. For each target mRNA, 20 reverse complement probes were designed in Serial Cloner (2.6.1) where a pair (P1 and P2) of split-initiator probes that hybridize and amplify a segment of target mRNA comprises two oligonucleotides, each 45 nt long (P1, 18-nt initiator sequence, 2-nt spacer, and 25-nt mRNA recognition sequence; P2, 25-nt mRNA recognition sequence, 2-nt spacer, and 18-nt initiator sequence). All probes were designed to have 40 to 75% GC content in their mRNA binding sites with a melting temperature ranging from 60 to 85°C and a minimal off-target complementarity (sequences that displayed >15-nt complementarity to nontarget mRNAs were excluded). HCR amplifiers were purchased from Molecular Instruments (Los Angeles, CA, USA). Sequences for HCR amplifiers B1, B2, and B3 used for combinatorial split-initiator probes have been described previously (19). Oligonucleotides were resuspended in nuclease-free water (Roth) at a final concentration of 1.0 μM and stored at −20°C.

10.1128/mSphere.00411-21.4 TABLE S4 HCR probes used in this study. Download Table S4, XLSX file, 0.01 MB. Copyright © 2021 Moreno-Velásquez and Pérez. 2021 Moreno-Velásquez and Pérez. https://creativecommons.org/licenses/by/4.0/ This content is distributed under the terms of the Creative Commons Attribution 4.0 International license.

### Sample preparation and hybridization chain reaction.

C. albicans overnight cultures were diluted 1:10 in fresh prewarmed YPD and incubated at 30°C for 35 min in a rotary shaker. To repress expression of the *PPZ1* gene, strain JCP_1128 was inoculated from YPD plates directly on yeast nitrogen base liquid medium containing cysteine (Roth) and methionine (Roth) at a final concentration of 2.5 mM and incubated for 1.5 or 3 h at 37°C with shaking (180 rpm). Cells were fixed in 4% paraformaldehyde for 45 min at room temperature. Cells were washed twice in PBS and resuspended in spheroplast buffer (100 mM potassium phosphate buffer [pH 7.0], 1.2 M sorbitol, 30 mM β-mercaptoethanol, and 40 μg/ml zymolyase 100T; preheated at 37°C) for 25 min at 37°C, with soft handshaking every 5 min. Spheroplasts were centrifuged at 3,700 rpm for 2 min and resuspended in washing buffer (100 mM potassium phosphate buffer [pH 7.0], 1.2 M sorbitol) twice. Spheroplasts (200 μl) were left to settle on polylysine-treated round slides in 12-well multiwell plates (Greiner) for 1 h at 4°C. Slides were fixed by the addition of 500 μl of ice-cold methanol for 6 min, then placed in 500 μl of ice-cold acetone for 30 s, and air dried. Wells were washed with 500 μl of PBS with Tween 20 (PBST) (1× PBS, 0.1% Tween 20) for 5 min.

Prehybridization and hybridization were carried out as described previously (19). Briefly, wells were incubated with 250 μl of probe hybridization buffer (50% formamide, 5× SSC [1× SSC is 0.15 M NaCl plus 0.015 M sodium citrate], 9 mM citric acid [pH 6.0], 0.1% Tween 20, 50 μg/ml heparin, 1× Denhardt’s solution, and 10% dextran sulfate) for 1 h at 37°C. The buffer was removed, and 250 μl of prewarmed hybridization buffer containing the probe (2 to 4 μl of 1 μM probe stock, depending on the abundance of the mRNA transcript) was added. Slides were incubated for 18 h at 37°C in darkness. After hybridization, slides were rinsed in 200 μl of prewarmed (37°C) washing buffer (50% formamide, 5× SSC, 9 mM citric acid [pH 6.0], 0.1% Tween 20, and 50 μg/ml heparin) to dilute the probes; the washing solution was removed, and the washes were repeated twice (5 min each) with prewarmed (37°C) washing buffer. After equilibration at room temperature, 200 μl of amplification buffer (5× SSC, 0.1% Tween 20, 10% dextran sulfate) was added to the slides and incubated for 30 min. In parallel, each fluorescently labeled hairpin (5 μl of 3 μM hairpin stock) was denatured at 95°C for 30 s and cooled to room temperature in the dark prior to dilution in 200 μl of fresh amplification buffer. For the hybridization chain reaction, slides were incubated in the hairpin-amplification buffer mixture at room temperature for 22 h in darkness. Samples were rinsed briefly in 300 μl of 5× SSCT (5× SSC and 0.1% Tween 20) and then washed in 5× SSCT for 5 min. To stain nuclei, slides were incubated with 4′,6′-diamidino-2-phenylindole (DAPI) (200 ng/ml in 5× SSCT) for 10 min. Wells were washed with 5× SSCT for 10 min and dried at room temperature prior to be mounted in ProLong diamond antifade mountant (Thermo Scientific). Samples were stored at 4°C for up to 2 months.

### Immunostaining.

After the HCR step and rinsing with 5× SSCT for 5 min, slides were incubated in blocking buffer (5% bovine serum albumin in TBS) for 30 min at room temperature in a humid chamber. A rat anti-α-tubulin (Bio-Rad) primary antibody was diluted 1:500 in blocking buffer and incubated for 1 h at room temperature in darkness. Slides were washed three times in TBS. Alexa Fluor 594-conjugated goat anti-rat secondary antibody (Thermo Scientific) was diluted 1:1,000 in blocking buffer containing DAPI (200 ng/ml), and slides were incubated for 1 h at room temperature in darkness. Samples were washed three times in TBS, dried, mounted in ProLong Diamond antifade mountant (Thermo Scientific), and stored at 4°C in darkness.

### Microscopy.

HCR cell imaging at high resolution was performed using a laser scanning confocal microscope (Leica, TCS SP5), which was mounted on an inverted microscope and equipped with photo multiplier tubes (PMT), hybrid GaAsP (HyD) detectors, and a 63× oil immersion objective (numerical aperture [NA], 1.4; HCX PL APO). Leica UV diode (405-nm), argon (488-nm), and HeNe (633-nm) lasers lines were used for fluorescence excitation. Simultaneous bright-field images were captured with a transmitted light detector. The laser intensity and exposure of the cells were kept to a minimum to reduce photobleaching. Confocal images were captured using Leica microsystem CMS software (LAS AF v.2.0.2) with or without zoom as series of short Z-stacks. The excitation and emission (Ex/Em) for fluorescent signals were used as follows: DAPI for nuclei (Ex/Em, 405/410-460 nm), hairpins-B1 conjugated to Alexa Fluor 546 (Ex/Em, 514/550-620 nm), hairpins-B2 conjugated to Alexa Fluor 647 (Ex/Em, 633/650-720 nm), and hairpins-B3 conjugated to Alexa Fluor 488 (Em/Ex, 488/500-540 nm).

For the simultaneous observation of three different mRNA transcripts and immunostaining coupled to HCR-FISH at high resolution, wide-field epifluorescence microscopy (Leica, DM16000 B), was used. A 63× oil plan fluor objective (NA, 1.4) was employed. The fluorescence intensity and exposure were kept to a minimum to reduce photobleaching. Excitation at 360 nm was used to visualize DAPI staining (nuclei). Excitation at 488, 514, and 633 nm was used to visualize the subcellular localization of mRNA transcripts that hybridized to B3, B1, and B2 hairpins, respectively. For HCR-FISH coupled to immunostaining, excitation at 561 nm was employed to detect α-tubulin. Maximum intensity projection processing of Z-stacks generated in this study was exported from LAS AF software and processed in FIJI (https://fiji.sc/).

### Flow cytometry.

Prior to flow cytometry, cells were diluted in PBS and further filtered through a 10-μm mesh to remove hyphal structures. Flow cytometry analyses were performed using a BD Accuri C6 Plus instrument (BD Biosciences). Approximately 50,000 cells were counted for every sample with the fluidic speed set at “slow.” The fluorescence signal was screened with the filter FL4 (670-nm-long pass filter) for transcripts hybridized to the hairpin B2. Flow cytometry data were gated and plotted using FlowJo (v.10). For relative quantification of mRNA transcripts, two gates were applied to the data: a first gate of forward scatter area (FSC-A) versus side scatter area (SSC-A) to select cells, and a second gate of FCS-A versus forward scatter height (FSC-H) to select single cells.

### Statistical analysis.

Statistical analysis was performed using GraphPad Prism software (v8.1). Data are presented as either means with interquartile range and 10th to 90th percentile or as means ± standard deviations (SD). Data sets were evaluated by Student’s *t* test (two-tailed, unpaired). *P* values of <0.05 were considered statistically significant. At least three biological replicates were carried out for every experiment included in this report.
