# Supplementary figures and images for: Imaging and Quantification of mRNA Molecules at Single-Cell Resolution in the Human Fungal Pathogen Candida albicans
Source: mSphere. 2021 Jul 7;6(4):e00411-21. doi: 10.1128/mSphere.00411-21 (PMC8386430; doi:10.1128/mSphere.00411-21)

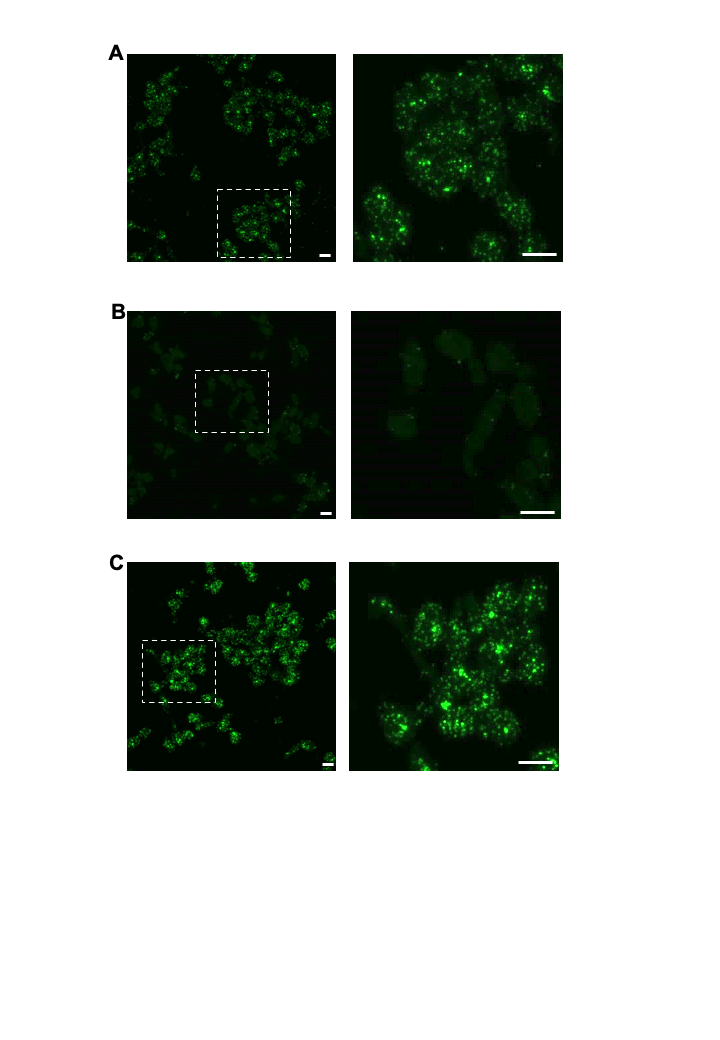

Supplement: FIG S1 [file msphere.00411-21-sf001.tif]

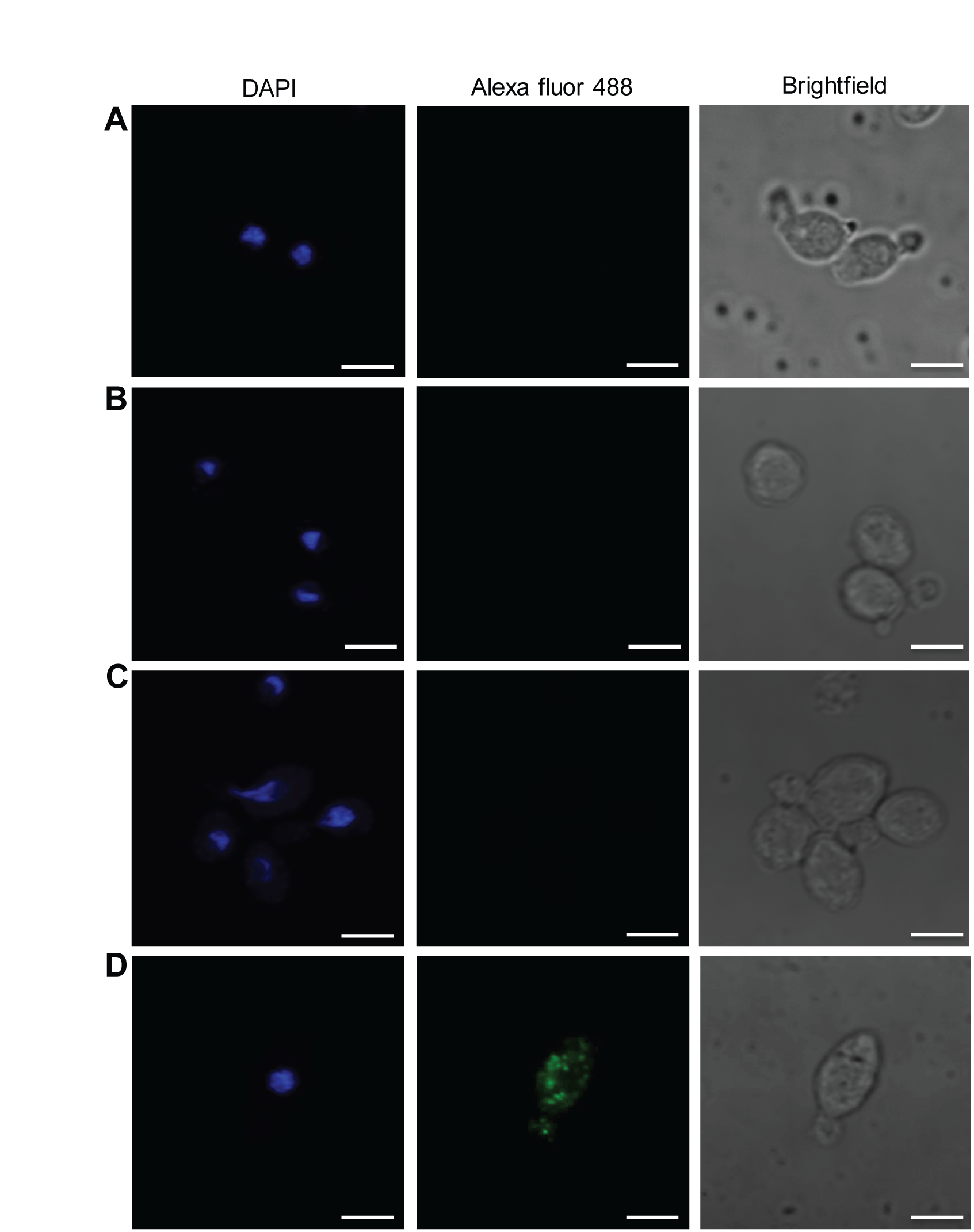

Supplement: FIG S2 [file msphere.00411-21-sf002.tif]

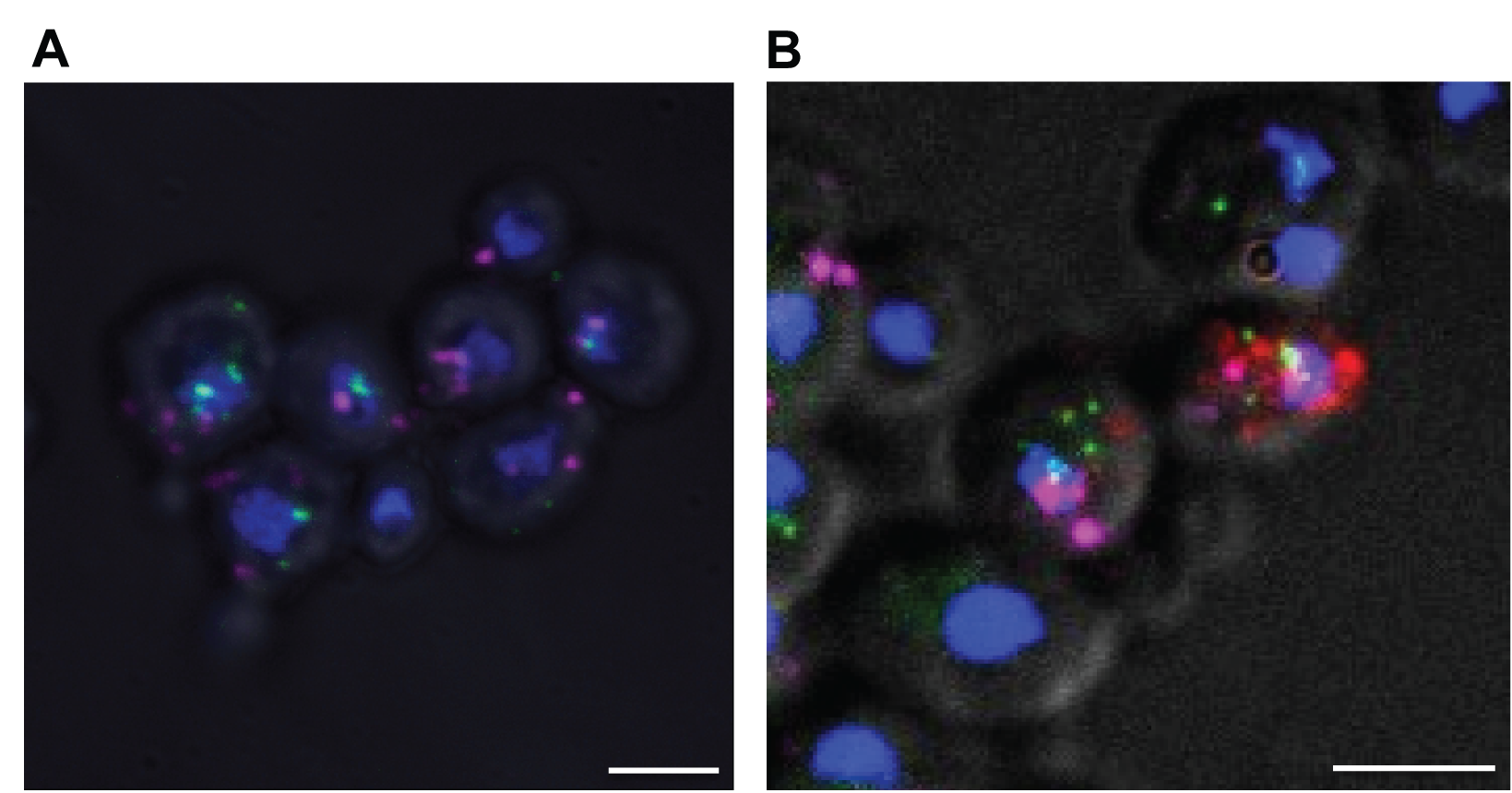

Supplement: FIG S3 [file msphere.00411-21-sf003.tif]
