# Supplementary material for: Imaging and Quantification of mRNA Molecules at Single-Cell Resolution in the Human Fungal Pathogen Candida albicans
Source: mSphere. 2021 Jul 7;6(4):e00411-21. doi: 10.1128/mSphere.00411-21 (PMC8386430; doi:10.1128/mSphere.00411-21)
Supplement: TABLE S1 [file msphere.00411-21-st001.pdf]

Table S1. Strains used in this study

Number	Strain name	Genotype	Source
JCP_520	SC5314	Wild-type reference strain	Gillum et al., 1984
JCP_ 1068	*rtg3*Δ/*rtg3*Δ	Δ*orf19.2315*/Δ*orf19.2315*	This study
JCP_1112	*ppz1*Δ/*PPZ1*	Δ*orf19.726*/*ORF19.726*	This study
JCP_1128	*ppz1*Δ/pMET-*PPZ1*	Δ*orf19.726*/*pMET*::*ORF726*	This study
JCP_401	SN250	ura3Δ::λimm434::URA3-IRO1 arg4::hisG his1::hisG leu2::hisG::CdHIS1 / ura3Δ::λimm434 arg4::hisG his1::hisG leu2::hisG::CmLEU2	Homann et al., 2009
JCP_248	pTDH3-*RTG3*/*RTG3*	ura3Δ::λimm434::URA3-IRO1 arg4::hisG his1::hisG leu2::hisG::CdHIS1 NAT::pTDH3-RTG3 / ura3Δ::λimm434 arg4::hisG his1::hisG leu2::hisG::CmLE	This study
TF_142	*rtg3*Δ/*rtg3*Δ	ura3Δ::λimm434::URA3-IRO1 arg4::hisG his1::hisG leu2::hisG rtg3Δ::CdHIS1 / ura3Δ::λimm434 arg4::hisG his1::hisG leu2::hisG rtg3Δ::CmLEU2	Homann et al., 2009
TF_37	*tye7*Δ/*tye7*Δ	ura3Δ::λimm434::URA3-IRO1 arg4::hisG his1::hisG leu2::hisG tye7Δ::CdHIS1 / ura3Δ::λimm434 arg4::hisG his1::hisG leu2::hisG tye7Δ::CmLEU2	Homann et al., 2009

References

Gillum AM, Tsay EYH, Kirsch DR. 1984. Isolation of the Candida albicans gene for orotidine-5′-phosphate decarboxylase by complementation of *S. cerevisiae* ura3 and E. coli pyrF mutations. Mol Gen Genet 198:179-182.

Homann OR, Dea J, Noble SM, Johnson AD. 2009. A phenotypic profile of the *Candida albicans* regulatory network. PLoS Genet 5:e1000783.
