# Supplementary material for: Imaging and Quantification of mRNA Molecules at Single-Cell Resolution in the Human Fungal Pathogen Candida albicans
Source: mSphere. 2021 Jul 7;6(4):e00411-21. doi: 10.1128/mSphere.00411-21 (PMC8386430; doi:10.1128/mSphere.00411-21)
Supplement: TABLE S2 [file msphere.00411-21-st002.pdf]

Table S2. Plasmids used in this study

Name	Resistance	Description	Source
pSFS5	Ampicillin	SAT1-FLIP	Sasse et al., 2011.
pADH34	Chloramphenicol	c-terminal 13xMyc	Hernday et al., 2010
pCJN542	Ampicillin	pTDH3-blank-SAT1	Nobile et al., 2008
pADH33	Ampicillin	pMET3-blank-SAT1	Lohse et al., 2013
pADH99	Ampicillin	HIS/FLP CAS9	Nguyen et al., 2017
pADH110	Ampicillin	Fragment A for cloning-free stitching of gRNA	Nguyen et al., 2017
pADH147	Ampicillin	Fragment B for cloning-free stitching of gRNA	Nguyen et al., 2017
JCP_1112	Ampicillin	pSFS5 + upstream/downstream ORF19.726	This study

References

Hernday AD, Noble SM, Mitrovich QM, Johnson AD. 2010. Methods Enzymol 470:737-758.
Lohse MB et al. 2013. Proc Natl Acad Sci USA 110:7660–7665.
Nguyen N, Quail MMF, Hernday AD. 2017. mSphere 2:e00149-17.
Nobile CJ et al.. 2008. Cell Microbiol 10:2180-2196.
Sasse C et al . 2011. PLoS One 6:e25623.
